# Supplementary material for: Time-series transcriptome analysis identified differentially expressed genes in broiler chicken infected with mixed Eimeria species
Source: Front Genet. 2022 Aug 8;13:886781. doi: 10.3389/fgene.2022.886781 (PMC9393255; doi:10.3389/fgene.2022.886781)
Supplement: Supplementary file 2 [file DataSheet1.ZIP › 7dpi_KEGG.Gsea.1625115961950/KEGG_MELANOMA.html]

Details for gene set KEGG\_MELANOMA[GSEA]

|  || Dataset | TMM\_7dpi\_gct\_format\_7dpi\_gct\_format.Class\_4dpi.cls #PC\_versus\_NC.Class\_4dpi.cls #PC\_versus\_NC\_repos |
| Phenotype | Class\_4dpi.cls#PC\_versus\_NC\_repos |
| Upregulated in class | 0 |
| GeneSet | KEGG\_MELANOMA |
| Enrichment Score (ES) | -0.5206105 |
| Normalized Enrichment Score (NES) | -1.703655 |
| Nominal p-value | 4.554771E-4 |
| FDR q-value | 0.08917324 |
| FWER p-Value | 0.2686 |
Table: GSEA Results Summary

  

Fig 1: Enrichment plot: KEGG\_MELANOMA      
 Profile of the Running ES Score & Positions of GeneSet Members on the Rank Ordered List

  

| SYMBOL | TITLE | RANK IN GENE LIST | RANK METRIC SCORE | RUNNING ES | CORE ENRICHMENT || 1 | CDKN1A | na | 843 | 0.575 | -0.0505 | No |
| 2 | FGF7 | na | 1167 | 0.467 | -0.0613 | No |
| 3 | HRAS | na | 1342 | 0.420 | -0.0613 | No |
| 4 | PDGFC | na | 2266 | 0.227 | -0.1306 | No |
| 5 | MET | na | 3037 | 0.122 | -0.1907 | No |
| 6 | PDGFD | na | 3313 | 0.085 | -0.2107 | No |
| 7 | MAP2K2 | na | 3435 | 0.070 | -0.2184 | No |
| 8 | FGF12 | na | 3438 | 0.069 | -0.2162 | No |
| 9 | FGFR1 | na | 4080 | -0.004 | -0.2696 | No |
| 10 | PDGFB | na | 4266 | -0.026 | -0.2842 | No |
| 11 | PDGFA | na | 4308 | -0.031 | -0.2865 | No |
| 12 | RAF1 | na | 5142 | -0.116 | -0.3521 | No |
| 13 | MAP2K1 | na | 5188 | -0.120 | -0.3517 | No |
| 14 | CDH1 | na | 6672 | -0.272 | -0.4662 | No |
| 15 | PDGFRA | na | 6935 | -0.300 | -0.4777 | No |
| 16 | MITF | na | 7022 | -0.311 | -0.4741 | No |
| 17 | AKT3 | na | 7033 | -0.312 | -0.4641 | No |
| 18 | E2F1 | na | 7190 | -0.328 | -0.4658 | No |
| 19 | KRAS | na | 7206 | -0.330 | -0.4556 | No |
| 20 | FGF9 | na | 7401 | -0.352 | -0.4596 | No |
| 21 | HGF | na | 7414 | -0.353 | -0.4483 | No |
| 22 | NRAS | na | 7474 | -0.358 | -0.4408 | No |
| 23 | CDK6 | na | 7686 | -0.382 | -0.4452 | No |
| 24 | RB1 | na | 8176 | -0.443 | -0.4707 | No |
| 25 | AKT1 | na | 8399 | -0.472 | -0.4729 | No |
| 26 | PIK3CG | na | 8478 | -0.481 | -0.4627 | No |
| 27 | PTEN | na | 8620 | -0.503 | -0.4570 | No |
| 28 | PDGFRB | na | 8726 | -0.517 | -0.4479 | No |
| 29 | PIK3R2 | na | 9597 | -0.645 | -0.4982 | Yes |
| 30 | IGF1R | na | 9854 | -0.683 | -0.4959 | Yes |
| 31 | E2F3 | na | 10031 | -0.717 | -0.4858 | Yes |
| 32 | MAPK3 | na | 10320 | -0.774 | -0.4830 | Yes |
| 33 | PIK3CB | na | 10335 | -0.777 | -0.4572 | Yes |
| 34 | CCND1 | na | 10432 | -0.796 | -0.4376 | Yes |
| 35 | PIK3CD | na | 10707 | -0.855 | -0.4308 | Yes |
| 36 | MDM2 | na | 10838 | -0.889 | -0.4108 | Yes |
| 37 | FGF10 | na | 10866 | -0.897 | -0.3819 | Yes |
| 38 | PIK3CA | na | 10920 | -0.912 | -0.3547 | Yes |
| 39 | PIK3R5 | na | 10941 | -0.918 | -0.3245 | Yes |
| 40 | PIK3R3 | na | 11045 | -0.949 | -0.3001 | Yes |
| 41 | CDKN2A | na | 11228 | -1.009 | -0.2803 | Yes |
| 42 | PIK3R1 | na | 11265 | -1.023 | -0.2478 | Yes |
| 43 | EGFR | na | 11415 | -1.085 | -0.2226 | Yes |
| 44 | FGF1 | na | 11593 | -1.171 | -0.1968 | Yes |
| 45 | BRAF | na | 11724 | -1.264 | -0.1638 | Yes |
| 46 | IGF1 | na | 11859 | -1.426 | -0.1255 | Yes |
| 47 | FGF19 | na | 11936 | -1.633 | -0.0752 | Yes |
| 48 | FGF13 | na | 12005 | -2.341 | 0.0004 | Yes |
Table: GSEA details [plain text format]

  

Fig 2: KEGG\_MELANOMA      
 Blue-Pink O' Gram in the Space of the Analyzed GeneSet

  

Fig 3: KEGG\_MELANOMA: Random ES distribution      
 Gene set null distribution of ES for **KEGG\_MELANOMA**
